# Association of immune cell traits with Parkinson’s disease: a Mendelian randomization study

**DOI:** 10.3389/fnagi.2024.1340110

**Published:** 2024-02-22

**Authors:** Zhiwei Song, Wangyu Li, Yupeng Han, Yiya Xu, Haiqi Ding, Yinzhou Wang

**Affiliations:** ^1^Department of Neurology, Fujian Provincial Hospital, Shengli Clinical Medical College of Fujian Medical University, Fuzhou, Fujian, China; ^2^Department of Pain Management, Fujian Provincial Hospital, Shengli Clinical Medical College of Fujian Medical University, Fuzhou, Fujian, China; ^3^Department of Anesthesiology, Fujian Provincial Hospital, Shengli Clinical Medical College of Fujian Medical University, Fuzhou, Fujian, China; ^4^Department of Orthopedic Surgery, The First Affiliated Hospital, Fujian Medical University, Fuzhou, Fujian, China; ^5^Fujian Key Laboratory of Medical Analysis, Fujian Academy of Medical Sciences, Fuzhou, Fujian, China

**Keywords:** Mendelian randomization, immune cells, Parkinson's disease, causal relationship, single nucleotide polymorphisms

## Abstract

**Background:**

Immunity and neuroinflammation play crucial roles in the pathogenesis of Parkinson’s disease (PD). Nonetheless, prior investigations into the correlation between immune inflammation and PD have produced varying results. Identifying specific immune cell phenotypes that are truly associated with PD is challenging, and the causal relationship between immune cells and PD remains elusive.

**Methods:**

This study conducted a comprehensive two-sample Mendelian randomization (MR) analysis, employing five distinct analytical approaches, to clarify the causal connection between immune cell characteristics and the risk of PD. Utilizing GWAS data, we investigated the causal relationship between 731 immune cell traits and PD. These immune cell phenotypes encompass absolute cell (AC) counts, median fluorescence intensity (MFI), and relative cell (RC) counts for B cells, cDCs, mature stage T cells, monocytes, myeloid cells, TBNK (T cells, B cells, and natural killer cells), and Tregs, as well as the logistic parameter (MP) for cDCs and TBNK.

**Results:**

The inverse variance weighted (IVW) analysis indicated that Myeloid DCs (*p* = 0.004), HVEM expression on CD45RA− CD4+ T cells (*p* = 0.007), CD62L− CD86+ Myeloid DCs (*p* = 0.015), and HLA DR expression on monocytes (*p* = 0.019) were associated with a reduced risk of PD. CD14+ CD16+ monocytes (*p* = 0.005), HLA DR+ NK cells within CD3− lymphocytes (*p* = 0.023), and CD28 expression on activated & secreting Tregs (*p* = 0.032) were associated with an increased risk of PD.

**Conclusion:**

This study establishes a causal link between immune cell phenotype and the pathogenesis of PD, identifying several specific immune cell characteristics associated with PD. This could inspire researchers to delve into the pathogenesis of PD at the cellular subtype level, and aid in the identification of potential pharmacological protein targets for PD.

## 1 Background

Parkinson’s disease (PD) is a progressive neurodegenerative disorder primarily characterized by the progressive loss of dopaminergic neurons in the substantia nigra and abnormal α-synuclein aggregation in Lewy vesicles (Bloem et al., 2021). In 2016, it was estimated that over 6 million individuals worldwide were affected by PD, a number that is projected to increase as the population ages (GBD 2016 Neurology Collaborators, 2019). The precise cause of PD remains elusive; however, research has indicated that neuroinflammation significantly contributes to PD’s development, with central nervous system (CNS)-resident immune cells (microglia, astrocytes) engaging in interactions with peripheral immune cells (macrophages, lymphocytes, etc.), leading to neuroinflammation, which markedly influences PD’s progression (Tansey et al., 2022).

Prior research has established the role of immune dysfunction, characterized by alterations in cytokine levels and irregularities in immune cell functionality, in the development of PD. Furthermore, PD shares numerous genetic risk factors with other autoimmune diseases, and several of the over 90 risk variants for PD identified by genome-wide association studies (GWAS) are related to immune function genes (Nalls et al., 2019). Furthermore, the identification of shared genetic variants in PD patients and those with other autoimmune diseases (Witoelar et al., 2017) further underscores the role of immunity in the pathogenesis of PD. Immune system dysregulation may occur in patients with autoimmune diseases, who are more likely to develop PD compared to the general population. A national epidemiological study involving 310,522 patients with autoimmune diseases revealed that 932 of these patients developed PD during the follow-up period (De Virgilio et al., 2016). Inflammatory cells (e.g., microglia, CD4+ and CD8+ T cells; Marogianni et al., 2020) and inflammatory factors (e.g., IL-1α, IL-2, IL-1β, TNF-α, IL-6, TGF-β, IFN-γ, and IL-9; Karpenko et al., 2018) serve as key mediators between the brain and the immune system and play a pivotal role in the pathogenesis of PD. In the brain tissue of PD patients, the levels of activated microglia and CD3+ T cells were found to be higher compared to those in control patients (Lin et al., 2019; Li et al., 2022). Studies have also demonstrated that PD patients exhibit elevated serum levels of pro-inflammatory cytokines, such as TNF, IFNγ, IL-1β, IL-6, IL-2, CXC-chemokine ligand 8 (CXCL 8), and CCL 2, which correlate with disease severity and disability (Wang et al., 2015). In recent research, it has been discovered that immune cells (NK cells, monocytes/macrophages, neutrophils, and T cells) from the peripheral system can penetrate the blood–brain barrier in individuals with PD (Huang et al., 2022). However, the role of immune cells in PD remains controversial; they may play a protective role in the acute phase of neuroinflammation in PD through the production of trophic factors and inhibition of inflammation (Hoogland et al., 2015), and in the chronic phase of neuroinflammation through inflammatory factors and autoantibodies that may exert toxic effects on neurons (Channer et al., 2023), which explains to some extent that one type of immune cell may lead to different conclusions in different studies about the pathogenesis of PD. While previous research has identified numerous associations between immune cells and PD, the exploration of the causal relationship between various immune cell phenotypes and the pathogenesis of PD is still pending. Furthermore, the specific phenotypic characteristics through which immune cells influence the course of PD have yet to be elucidated.

Mendelian Randomization (MR) employs variations in genetics as a pivotal instrument for evaluating the causal relationships between exposures and subsequent outcomes (Bowden and Holmes, 2019), and is primarily employed for etiological inference in epidemiology, aiding researchers in reducing bias in experimental results and in drawing conclusions about causality (Emdin et al., 2017). Although randomized controlled trials (RCTs) are effective tools for causal inference, their implementation can be resource-intensive and is often limited by sample size and ethical issues. However, MR offers advantages such as lower cost, reduced confounding, and diminished reverse causal inference compared to observational study (Sekula et al., 2016), and is thus regarded as a “natural” RCT that aids in mitigating the traditional bias inherent in observational studies (Emdin et al., 2017). The primary objectives of this study involve assessing the causal relationship between various immune phenotypes and PD, while the secondary objectives include identifying potential key immune cell phenotypes, deepening the understanding of PD pathogenesis, and providing new directions and strategies for PD treatment and drug development.

## 2 Materials and methods

### 2.1 Study design

**Figure 1:**
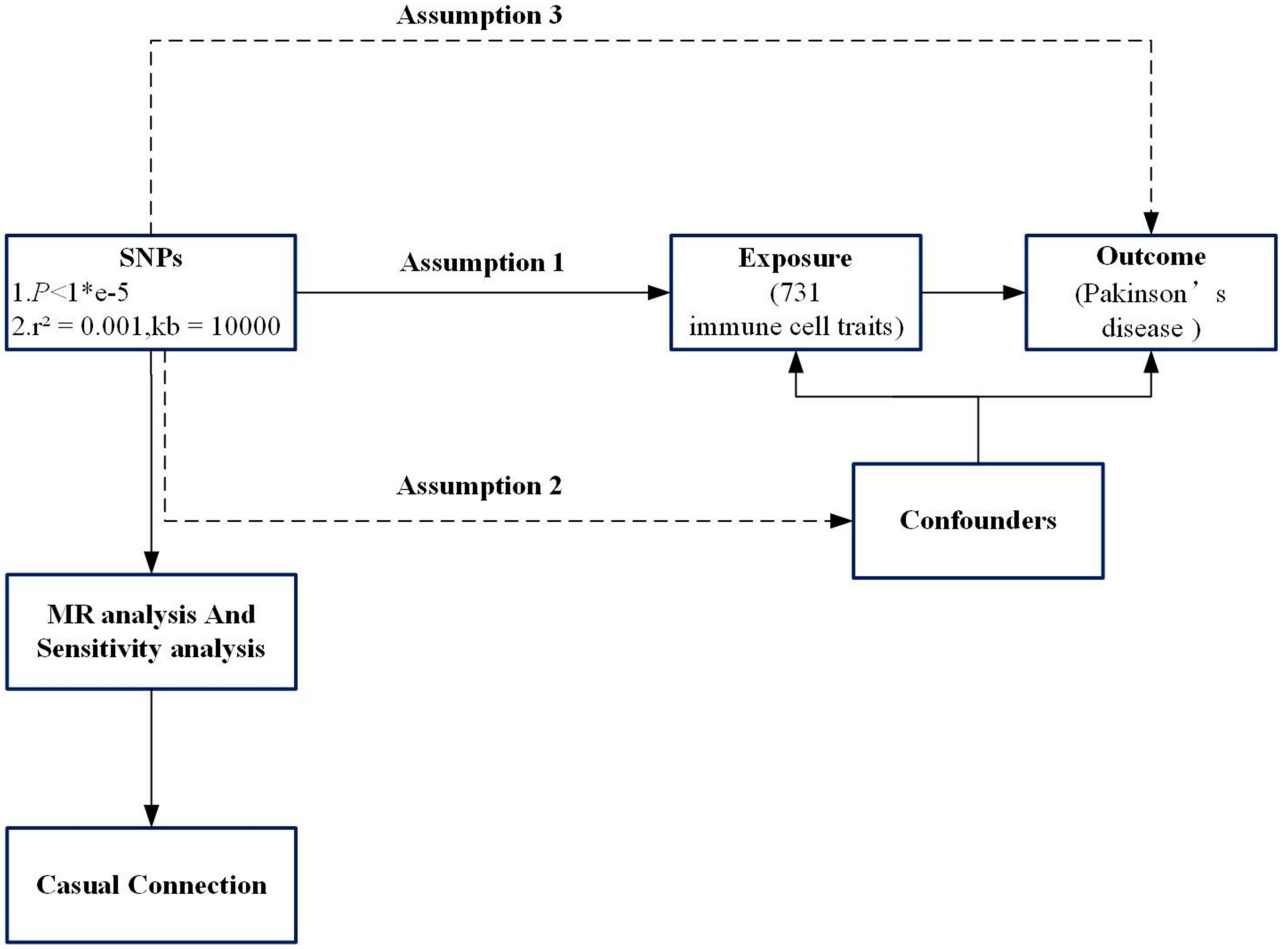
Flowchart of the study. Assumption 1, the exposure (immune phenotype) maintains a robust association with the chosen single-nucleotide polymorphisms (SNPs); Assumption 2, SNPs are not influenced by external confounding factors; Assumption 3, SNPs do not have a direct relationship with the outcome (PD), their causal connection is exclusively through the immune phenotype. SNP, Single-nucleotide poly-morphism; MR, Mendelian randomization.

Throughout its duration, the study was conducted in strict adherence to the STROBE-MR guidelines (Skrivankova et al., 2021). In this study utilizing two-sample Mendelian Randomization, loci of single nucleotide polymorphism (SNP) from the database of genome-wide association analysis (GWAS) were employed as instrumental variables for examining the influence of risk between immune phenotypes and PD. A total of 731 immune cell characteristics were defined as exposure factors in relation to the occurrence of PD, constituting the study outcome. The analysis utilizing MR adheres to three foundational principles: firstly, the exposure (immune phenotype) maintains a robust association with the chosen SNPs; secondly, these SNPs are not influenced by external confounding factors; and thirdly, while these polymorphisms do not have a direct relationship with the outcome (PD), their causal connection is exclusively through the immune phenotype (Burgess and Labrecque, 2018). The design and hypotheses and flow chat of the MR study are outlined in Figure 1.

### 2.2 Source of exposure data

Publicly available immune trait data were obtained from Valeria Orrù’s study (Orrù et al., 2020), encompassing a total of 731 immunophenotypes. This dataset includes a total of 118 cell counts, 389 median fluorescence intensities (MFIs) associated with surface antigens, in addition to 32 cell morphology parameters, and 192 relative counts, which refer to the ratio among different cells. This data were sourced from a cohort of 3,757 Sardinians, encompassing 20,143,392 SNPs (Orrù et al., 2020).

### 2.3 Collection of outcome data

Extensive GWAS genetic data were obtained from the International Parkinson’s Disease Genomics Consortium (IPDGC), encompassing 7.8 million SNPs from 37,700 cases, 18,600 proxy cases (individuals without PD yet having a familial history of the condition), and 1.4 million controls (Nalls et al., 2019). This constitutes one of the largest GWAS studies to date on PD patients of European ancestry, with data from the United Kingdom Biobank being openly accessible.

### 2.4 Identification of SNPs

The causal relationship between immunophenotype and PD was assessed. The specific screening steps included using the TwoSampleMR package to extract relevant SNPs (Woolf et al., 2022). SNPs that exhibited significant differences across the genome (*p* < 1*e−5) (VanderWeele et al., 2014) were selected and tested for linkage disequilibrium (*r*^2^ = 0.001, kb = 10,000), followed by the elimination of linkage disequilibrium SNPs and the removal of echo sequences. To reduce bias due to weak instruments, SNPs displaying *F*-statistics (a measure of the SNP’s robustness; Burgess et al., 2016; indicative of the SNP’s strength) lower than 10 were omitted.

### 2.5 Statistics and sensitivity analyses

In this study, R 4.3.2 software (Yavorska and Burgess, 2017) was primarily utilized to conduct Inverse Variance Weighted (IVW), MR Egger, Weighted Median, Simple Mode, and Weighted Mode analyses. The IVW method served as the principal analytical approach to ascertain the causal relationship between immunophenotypes and PD. Additionally, reverse Mendelian randomization was employed to investigate the influence of PD onset on immunophenotype (Davey Smith and Hemani, 2014). The heterogeneity among the estimated effects of SNPs was assessed by calculating both the *Q*-value and *p* value, following Cochran’s method. In the presence of heterogeneity, causal inference was conducted using the IVW random effects model (Burgess et al., 2017). The MR-Egger intercept examination and the MR-PRESSO approach were applied to investigate horizontal pleiotropy. Subsequently, a Leave-one-out sensitivity analysis (Burgess and Thompson, 2017) was performed to evaluate the impact of individual SNPs on the overall causal effect. Moreover, funnel plots and scatter plots were produced to visually illustrate the possible presence of horizontal pleiotropy.

## 3 Results

### 3.1 SNP screening and MR analysis results

Following the aforementioned steps, which involved matching data on immunophenotypes and PD and excluding SNPs with low F-statistics (< 10), a total of 147 SNPs were ultimately included in the study.

### 3.2 Exploration of the role of immunophenotype on the pathogenicity of PD

**Figure 2:**
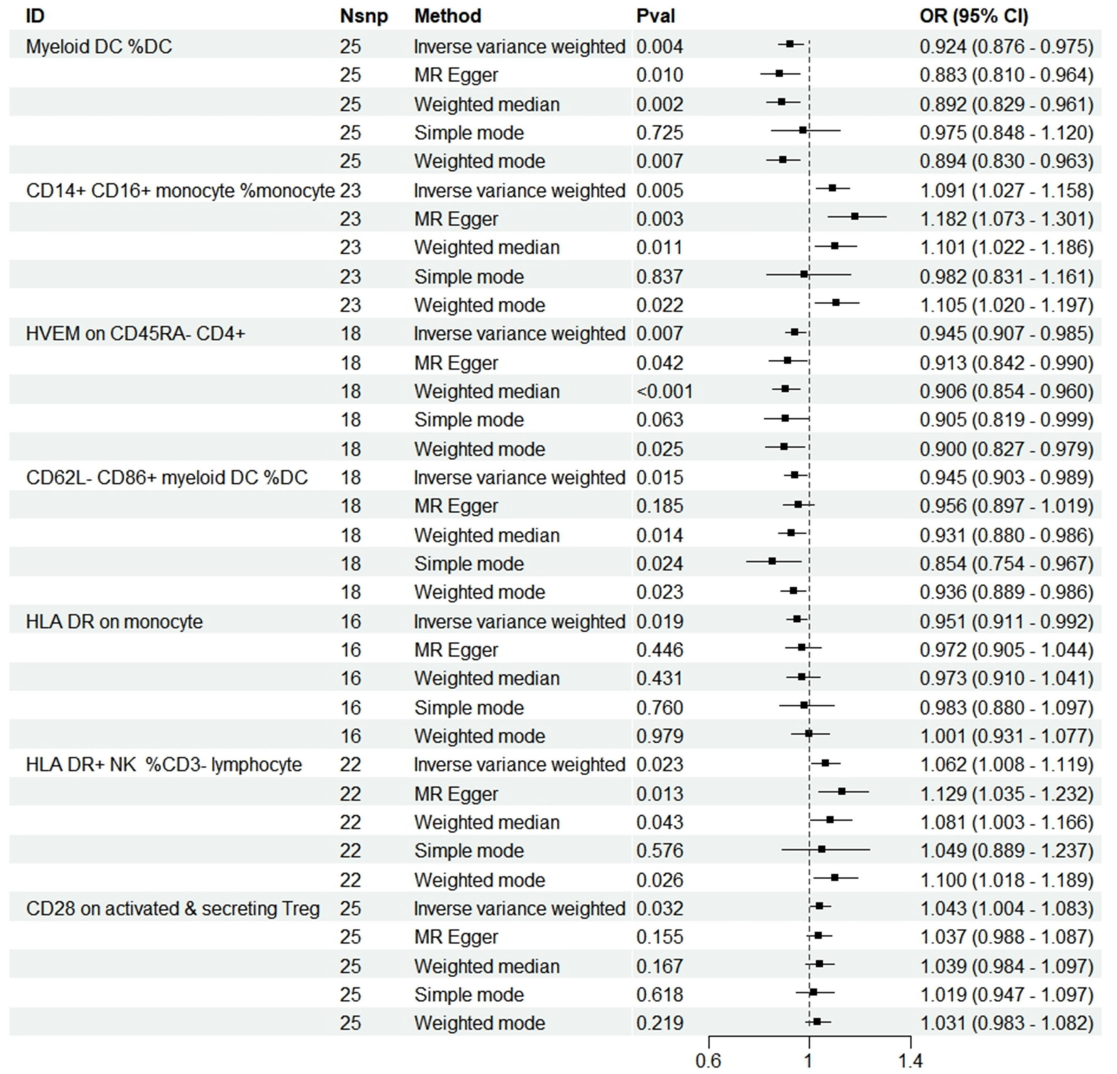
Forest plots showed the causal associations between immune cell traits and PD by using different methods. SNP, Single-nucleotide poly-morphism; OR, Odd radio; and CI, Confidence interval.

At a significance threshold of *p* < 0.05 using the IVW method, it was observed that Myeloid DC within the DC (cDC group), CD14+ CD16+ monocytes (monocyte group), HVEM on CD45RA− CD4+ T cells (Maturation stages of T cell group), CD62L− CD86+ myeloid DC within the DC (cDC group), HLA DR on monocytes (monocyte group), HLA DR+ NK cells in CD3− lymphocytes (TBNK group), and CD28 on activated & secreting Treg cells (Treg group) demonstrated significant associations with PD (Figure 2). Results derived from five distinct MR methods are presented in Figure 2. MR analyses employing a random-effects model with the IVW method yielded the following results: myeloid DC of DC (OR = 0.924, 95%CI: 0.876–0.975, *p* = 0.004), HVEM on CD45RA− CD4+ T cell (OR = 0.945, 95%CI: 0.907–0.985, *p* = 0.007), CD62L− CD86+ myeloid DC of DC (OR = 0.945, 95%CI: 0.903–0.989, *p* = 0.015), and HLA DR on monocyte (OR = 0.951, 95%CI: 0.911–0.992, *p* = 0.019) indicated a protective effect against reduced PD risk. The CD14+ CD16+ monocyte (OR = 1.091, 95%CI: 1.027–1.158, *p* = 0.005), HLA DR+ NK cell in CD3− lymphocytes (OR = 1.062, 95%CI: 1.008–1.119, *p* = 0.023), and CD28 on activated & secreting Treg (OR = 1.043, 95%CI: 1.004–1.083, *p* = 0.032) were identified as being associated with an increased risk of PD. Four additional analytical methods—MR Egger, Weighted Median, Simple Mode, and Weighted Mode—were employed to ascertain the stability of the results, yet the Simple Mode method did not substantiate a causal relationship for Myeloid DC within DCs (OR = 0.975, 95% CI: 0.848–1.120, *p* = 0.725), CD14+ CD16+ Monocytes (OR = 0.982, 95% CI: 0.831–1.161, *p* = 0.837), HVEM on CD45RA− CD4+ T cells (OR = 0.905, 95% CI: 0.819–0.999, *p* = 0.063), or HLA DR+ NK cells within CD3− lymphocytes (OR = 1.049, 95% CI: 0.889–1.237, *p* = 0.576) in relation to PD. Furthermore, the MR Egger method also failed to demonstrate a causal relationship between CD62L− CD86+ Myeloid DCs within DCs (OR = 0.956, 95% CI: 0.897–1.019, *p* = 0.185) and PD, additionally, numerous analyses did not corroborate a causal relationship between HLA DR+ NK cells within CD3− lymphocytes and CD28 on activated and secreting Tregs in relation to PD, but the IVW results were significant, with no detected pleiotropy or heterogeneity, and the outcomes of all five analytical methods aligned in the same direction, suggesting a positive result overall (Birney, 2022; Figure 2).

Concurrently, a reverse Mendelian randomization analysis was carried out to evaluate the influence of PD manifestation on immunophenotypes. Although the results varied significantly across different analysis methods, they failed to satisfy both the MR-Egger intercept test and the MR-PRESSO method, indicating the presence of horizontal pleiotropy and leading to an unreliable result.

### 3.3 Sensitivity analyses

**Figure 3:**
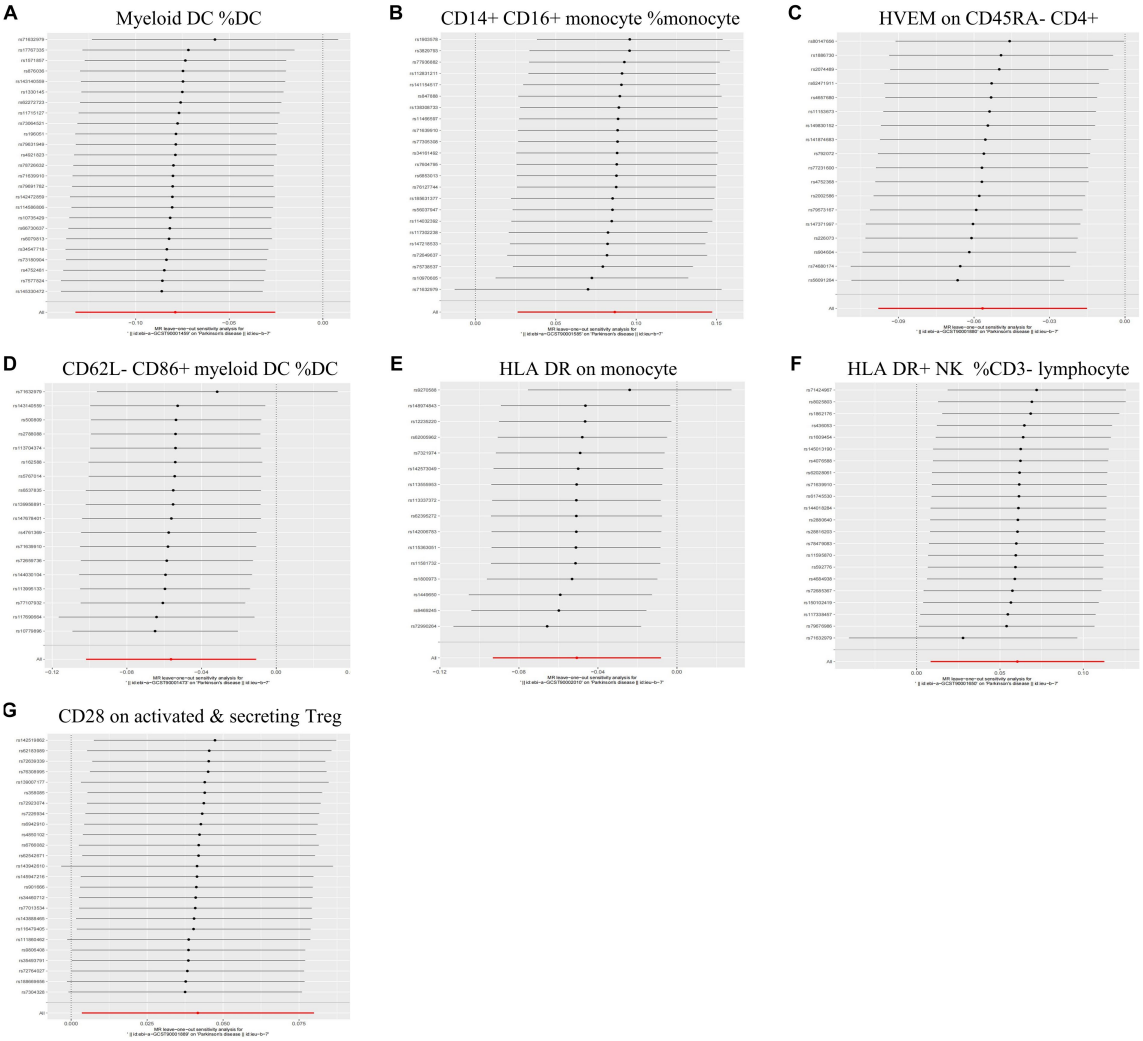
Leave-one-out analysis of each immunophenotype on the risk of PD. The error line represents a 95% confidence interval. **(A)** Myeloid DC of DC; **(B)** CD14+ CD16+ monocyte of monocyte; **(C)** HVEM on CD45RA− CD4+ T cell; **(D)** CD62L− CD86+ myeloid DC of DC; **(E)** HLA DR on monocyte; **(F)** HLA DR+ NK of CD3− lymphocyte; and **(G)** CD28 on activated & secreting Treg.

**Figure 4:**
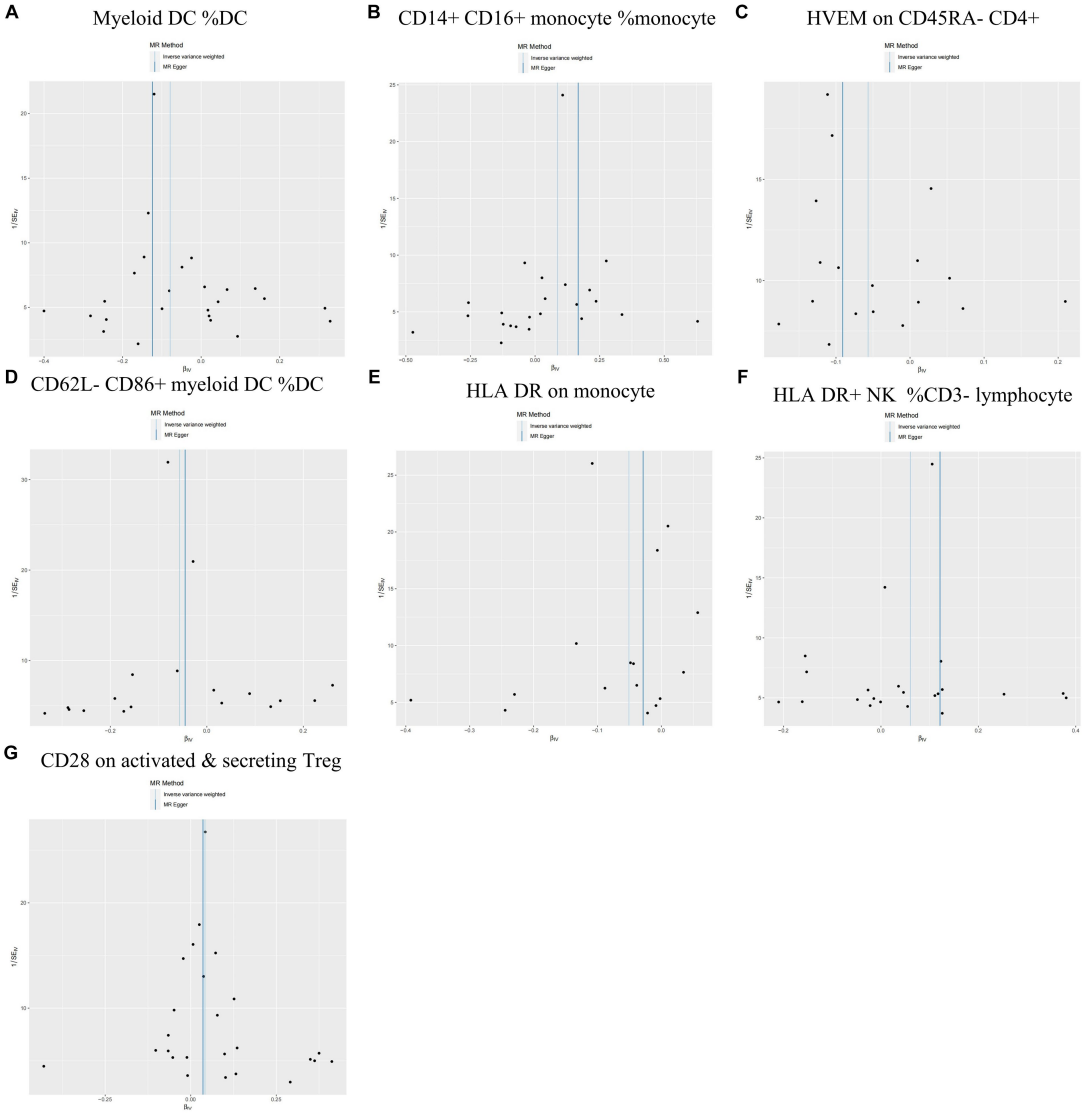
Funnel plots are utilized in sensitivity analysis to examine the causal relationship between immunophenotype and the risk of PD. **(A)** Myeloid DC of DC; **(B)** CD14+ CD16+ monocyte of monocyte; **(C)** HVEM on CD45RA− CD4+ T cell; **(D)** CD62L− CD86+ myeloid DC of DC; **(E)** HLA DR on monocyte; **(F)** HLA DR+ NK of CD3− lymphocyte; and **(G)** CD28 on activated & secreting Treg.

**Figure 5:**
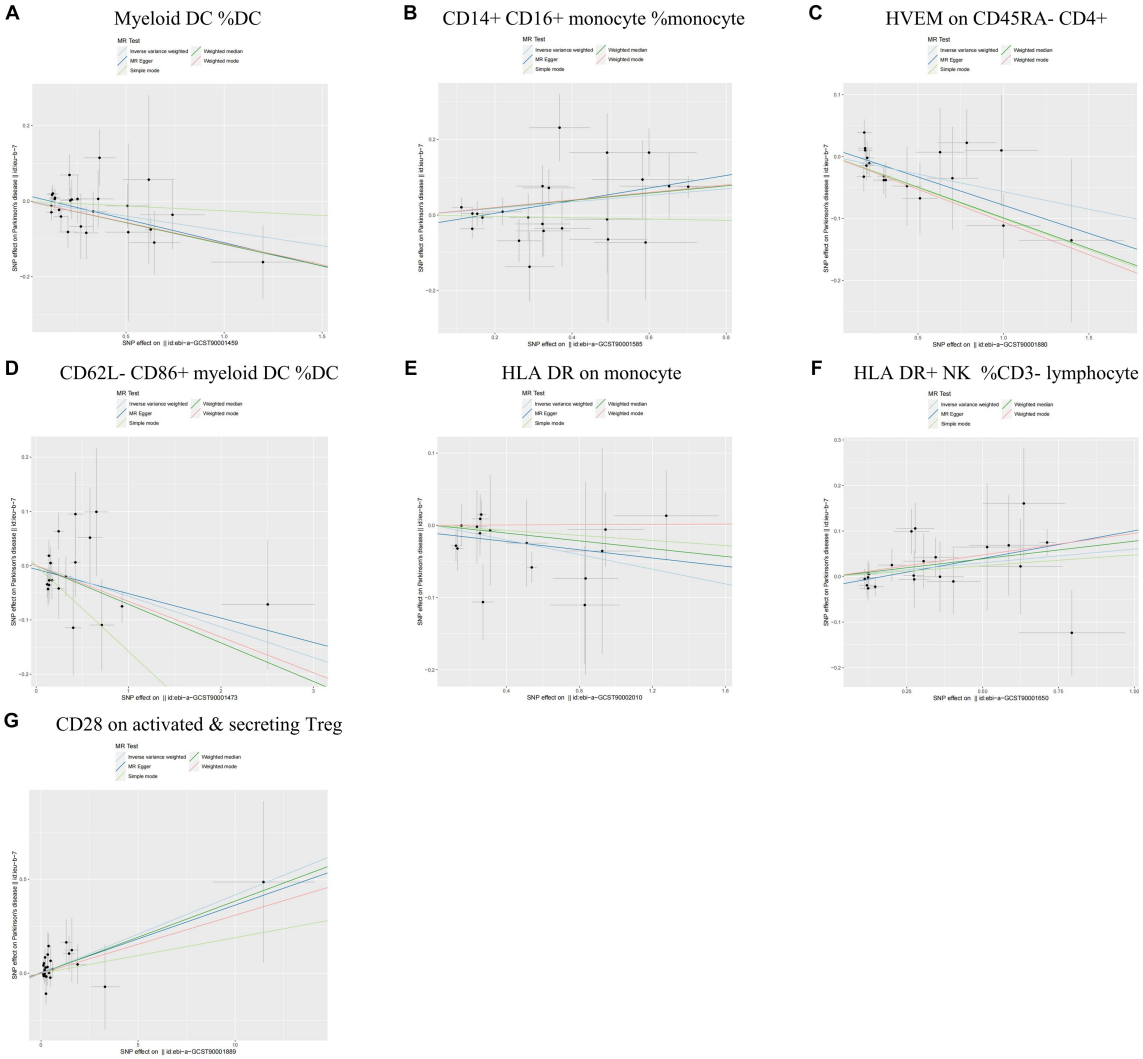
Scatter plots of sensitivity analysis for the causal relationship between immunophenotype and the risk of PD. **(A)** Myeloid DC of DC; **(B)** CD14+ CD16+ monocyte of monocyte; **(C)** HVEM on CD45RA− CD4+ T cell; **(D)** CD62L− CD86+ myeloid DC of DC; **(E)** HLA DR on monocyte; **(F)** HLA DR+ NK of CD3− lymphocyte; and **(G)** CD28 on activated & secreting Treg.

During the analysis of these seven causal links, all *p* values derived from Cochran’s *Q* test for heterogeneity surpassed the significance level set at 0.05. This suggests the absence of substantial heterogeneity among the SNPs associated with PD, as detailed in Table 1. Additionally, our MR-Egger-intercept analysis revealed the absence of significant directional pleiotropy for each immunophenotype (*p* > 0.05). This implies included SNPs were not associated with the outcome via confounding factors. Causal effects, as estimated by the MR-PRESSO method, remained significant both prior to and following outlier correction. Furthermore, global analyses did not uncover any evidence of horizontal pleiotropy (global test *p* > 0.05). The leave-one-out analysis illustrates that none of the individual SNP exert a significant effect on the overall causal estimate, thereby bolstering the reliability of the MR results (Figure 3). Additionally, the funnel plot exhibited a predominantly symmetrical distribution of causal effects, indicating minimal bias (Figure 4). Scatter plots showed that the regression lines illustrating the connection between immunophenotypes and PD risk, as obtained from different analytical approaches, were broadly consistent (Figure 5).

**Table 1:** Sensitivity analysis for immune cell traits vs. PD by MR analysis.

Exposure	Horizontal pleiotropy		Heterogeneity	MR-PRESSO global test
	MR egger-interpreter	MR egger-interpreter	Cochran’s *Q*	*p* value
		*p* value	*p* value	
Myeloid DC %DC	0.014	0.209	0.760	0.720
CD14+ CD16+ monocyte %monocyte	0.029	0.057	0.375	0.271
HVEM on CD45RA− CD4+	0.013	0.344	0.546	0.569
CD62L− CD86+ myeloid DC %DC	0.007	0.614	0.315	0.377
HLA DR on monocyte	0.011	0.463	0.6037	0.516
HLA DR+ NK % CD3− lymphocyte	0.021	0.104	0.769	0.580
CD28 on activated & secreting Treg	0.004	0.697	0.469	0.575

OR: Odd radio; CI: Confidence interval.

## 4 Discussion

Peripheral immunity may contribute to PD pathogenesis through mechanisms including inflammatory responses, cellular infiltration, dysregulation of lymphocyte subsets, and autoimmune responses. The findings from the two-sample Mendelian randomization indicated that CD14+ CD16+ monocytes, HLA DR+ NK cells in CD3− lymphocytes, and CD28 on activated & secreting Tregs were positively correlated with an increased risk of PD. Furthermore, it was observed that Myeloid DCs, HVEM on CD45RA− CD4+ T cells, CD62L− CD86+ Myeloid DCs, and HLA DR on monocytes exerted a protective causal effect against the development of PD. Sensitivity analyses and quality control affirmed the robustness of these findings. Exposure factors in this study, derived from peripheral blood immune cell phenotypes as detected by flow cytometry, partially substantiate the causal relationship between peripheral immunity and PD’s development.

In the evolving process of immune-brain crosstalk, it appears that the systemic immune response constitutes an integral component of the pathogenesis of PD, wherein myeloid-derived dendritic cells (Myeloid DCs) assume a pivotal role (Tansey et al., 2022). In PD, a subset of myeloid-derived dendritic cells, which have metastasized from the peripheral blood, may infiltrate the brain, harvesting cerebrospinal fluid (CSF) brain antigens in the choroid plexus or meninges. This process induces immune cells to adopt dysregulated phenotypes, thereby contributing to an inflammatory environment that plays a pathological role in neurodegeneration (Bossù et al., 2015). A Ciaramella et al. observed that PD patients showed significantly lower levels of peripheral blood Myeloid DCs compared to control subjects, with the number of peripheral blood Myeloid DCs being negatively correlated with the severity of motor symptoms (Goldeck et al., 2016). This is consistent with our findings, suggesting that Myeloid DC might play a protective role in the pathogenesis of PD. We hypothesized that a reduction in the level of peripheral blood Myeloid DC is associated with increased recruitment of this cell from the circulatory system to the brain, potentially explaining the more active cellular infiltration and inflammatory response in the brain of PD patients. Consequently, peripheral blood myeloid cells reflect, to some degree, the extent of neuroinflammation and may serve as a potential biomarker for evaluating PD’s progression.

Our results suggest that an elevation in the levels of CD14 + CD16+ monocyte subsets is associated with an increased risk of PD. The peripheral immune system, particularly its monocytes, has been implicated in the neuroinflammation associated with PD. α-Synuclein, identified as a damage-associated molecular pattern (DAMP) released by neurons, contributes to the *in vivo* activation of monocytes. This activation prompts the monocytes to produce pro-inflammatory mediators (Grozdanov et al., 2019). Monocytes, a heterogeneous group of cells, are primarily characterized by variations in the expression of CD14 and CD16 molecules. In PD patients, alterations in the phenotype and function of these monocytes are evident, as indicated by a rise in the number of CD14 + CD16+ monocyte subpopulations in the blood. This particular subpopulation may exhibit high expression of TLR10 (da Rocha Sobrinho et al., 2021). Emerging data suggest a correlation between the frequency of CD14 + CD16 + TLR10+ monocytes and the severity of PD (da Rocha Sobrinho et al., 2021), these observations align with the results of our findings. The role of CD14 + CD16+ monocytes in PD continues to be a topic of debate in the scientific community. Conversely, Schlachetzki et al. (2018) observed no change in the frequency of CD14 + CD16+ monocyte subpopulations in PD patients compared to healthy controls. However, Grozdanov et al. (2014) reported a decrease in the number of CD14 + CD16+ monocytes in PD patients. Variations in the age of onset, duration, and severity of PD among patient populations, as well as the overall sample sizes of the studies, may contribute to the divergent findings. The use of MR in our study helped to partially negate the effects of confounding factors and reverse causality.

CD4+ T cells potentially play a critical role in neurodegeneration associated with PD. Prior research has demonstrated that CD4-deficient mice exhibit attenuated dopaminergic neuronal death due to α-synuclein overexpression. IFN-γ-producing CD4+ T cells are crucial for CNS myeloid MHCII responses and TH nerve neuronal loss attributed to α-synuclein overexpression (Deng et al., 2021). Additionally, it has been observed by some researchers that CD4+ and CD8+ T cells infiltrate the brain parenchyma in brain tissue samples from PD patients, as well as in mouse models of PD (Sanchez-Guajardo et al., 2010). A meta-analysis of 21 case–control trials (Jiang et al., 2017), encompassing 943 PD cases, indicated that the number of peripheral blood CD4+ T cell subsets is reduced in PD patients, aligning with our findings. Furthermore, HVEM, a member of the tumor necrosis factor receptor (TNFR) superfamily (Aubert et al., 2021), inhibits T-cell proliferation when bound to the B and T lymphocyte attenuator (BTLA). This inhibition occurs through the phosphoacyl esterase SHP-1 and SHP-2 downstream signaling pathways, ultimately suppressing T cell activation and immune responses (Deng et al., 2021). Zhong et al. provided evidence that elevated HVEM expression in CD4+ T cells could act as a protective factor in myasthenia gravis. Our study echoes these findings, proposing that HVEM expression in CD45RA− CD4+ T cells might play a protective role in the development of PD by attenuating the immune response.

Prior research has demonstrated that PD patients exhibit reduced levels of peripheral blood myeloid DC (Ciaramella et al., 2013). Conversely, our current study reveals that CD62L− CD86+ myeloid DC within the dendritic cell (DC) genus play a protective role in preventing PD. DCs are critical in initiating T cell responses and coordinating immune responses, while CD86, a co-stimulatory molecule, is believed to promote the Th2 response. *In vitro* studies have shown that IFN-β upregulates CD86 expression on DCs and inhibits the Th1 response via these cells (Tekguc et al., 2021). DCs are crucial in initiating T cell responses and coordinating immune reactions. Peng et al. (2022) reported that extracellular vesicles derived from mesenchymal stromal cells may suppress the Th2 response in allergic rhinitis patients by inhibiting CD86 expression on DCs. Furthermore, numerous investigators have noted diminished levels of CD86 expression in the blood DCs of individuals with multiple sclerosis (MS), suggesting that CD86+ myeloid DCs may have a protective effect in MS (Huang et al., 2001). Limited research exists on the association between CD62L− CD86+ myeloid DC of DC and PD; it is hypothesized that these cells may mitigate PD risk by modulating the Th2 response.

Parkinson’s disease have identified changes linked to innate immune abnormalities in CSF (Wijeyekoon et al., 2020a,b) and peripheral blood, notably in monocyte subtypes and their marker expression (Su et al., 2022). Our results indicate that HLA-DR expression on monocytes within the Monocyte genus plays a protective role in the pathogenesis of PD. In a study by Wijeyekoon et al. (2020a,b), it revealed no significant variance in the overall count of serum monocytes between PD patients and controls, however, the phenotype of these monocytes had changed. The surface expression levels of monocyte HLA-DR were found to be associated with better cognitive function, semantic fluency, and motor function. Therefore, reduced levels of HLA-DR may result in ineffective clearance of pathological proteins, potentially triggering neuronal dysfunction and ultimately leading to cognitive and motor deficits (Wijeyekoon et al., 2020a,b). However, this finding contrasts with some animal models of PD, where increased HLA-DR has shown negative effects (Harms et al., 2013). The expression of the MHCII complex plays a crucial role in α-synuclein-induced microglia activation within the pathological framework of PD (Harms et al., 2013), with HLA-DR being a component of the MHCII complex. Inconsistent findings concerning HLA-DR might stem from microglial activation triggered by HLA-DR during acute toxin/protein injections or pathology in PD animal models, where the deleterious effects of pro-inflammatory cytokine release surpass the benefits of enhanced pathological protein clearance. Conversely, in chronic clinical disease scenarios, the benefits of increased antigen presentation and α-synuclein clearance by HLA-DR may exceed its potential deleterious impacts.

Natural killer (NK) cells, a crucial component of innate immunity, modulate neuroinflammation when infiltrated into the CNS (Earls and Lee, 2020). Research conducted by Sun et al. (2019) demonstrated that the proportion of NK cells was significantly higher in PD patients compared to controls. Additionally, individuals with peripheral blood NK cell levels exceeding the reference range had a significantly increased risk of developing PD. This aligns with our observation that HLA-DR+ NK cells in CD3− lymphocytes could potentially increase the risk of developing PD. In a mouse model of amyotrophic lateral sclerosis (ALS), NK cells within the spinal cord prompt microglia to transition to the M1 type, releasing inflammatory mediators via interferon γ (IFN-γ) (Garofalo et al., 2020). Additionally, HLA-DR+ NK cells have been linked to substantial IFN-γ production (Erokhina et al., 2018). IFN-γ could potentially exacerbate neuroinflammation in the mid and late stages of PD, resulting in further deterioration of neuronal damage (Panagiotakopoulou et al., 2020). Therefore, we hypothesize that the heightened risk of PD development due to HLA-DR+ NK cells in CD3− lymphocytes might be linked to neuroinflammation, potentially mediated by elevated levels of inflammatory cytokines like IFN-γ.

Prior research has shown that CD4 + CD25 + Foxp3+ regulatory T cells (Treg) uphold immune homeostasis via their immunosuppressive action on effector T cells (Teff) (Ohkura and Sakaguchi, 2020). Additionally, observations that PD patients commonly exhibit a decreased ability of Treg to suppress Teff (DeMaio et al., 2022), indicate that the development of PD may lead to a significant reduction in Treg’s ability to suppress inflammation. However, there remains a debate in the literature regarding how the ratio of peripheral blood Treg changes in PD patients. Findings by Kustrimovic et al. (2018) indicated that the number of circulating Treg cells was significantly lower in PD patients, with CD4+ T cells showing a preferential differentiation toward the Th1 lineage. On the other hand, a study conducted by Cen et al. (2017) showed that there was no significant disparity in Treg ratios between the PD group and the control group; however, the Treg/Th17 ratio was significantly higher in female PD patients compared to female controls. These discrepancies could be attributed to biases arising from differences in the race of the PD patients included in the study, the severity of the disease, and the overall sample size. Additionally, CD28 receptor, expressed on Treg cells, acts as a co-stimulatory molecule, providing a secondary signal crucial for full T cell activation. In the hαSyn mouse model of PD, Treg activation and expansion occur through binding to the CD28 receptor via a hyper-excitable anti-CD28 monoclonal antibody (CD28SA). This process attenuates neuroinflammatory responses and mitigates dopaminergic neuronal degeneration (Badr et al., 2022), evidencing neuroprotective effects. Notably, our results indicate that CD28 on activated and secreting Treg may increase the risk of developing PD. We contend that Mendelian randomization analysis, which employs genetic variants as instrumental variables, is not influenced by extraneous factors such as the environment, since these variables are established at the time of conception. Therefore, the results of MR are indicative of the effects of lifetime perturbations of risk factors (Larsson et al., 2023), potentially leading to a distinct acute effect compared to that observed in animal models with CD28. In other words, the impact of enhanced Treg immunosuppression, resulting from increased CD28 signaling in PD, may vary during different stages of PD onset and progression. Enhanced Treg function in the initial stages of the disease might aid in controlling the brain’s inflammatory response, potentially slowing PD progression. Conversely, prolonged Treg activation could lead to excessive immune suppression, impacting neural tissue clearance and repair, and indirectly advancing disease progression. Future research should focus on conducting more prospective studies to further explore the molecular role of CD28 on activated and secreting Treg in PD and other neuroinflammatory diseases.

The current study found insufficient statistical evidence to support an inverse relationship between immune cell phenotype and PD. While the IVW method indicated a correlation and differential significance between PD development and various immune phenotypes, the pronounced multiplicity of these associations contravenes the exclusionary assumptions of MR. This indicates that certain SNPs might influence changes in the immune phenotype via confounding factors other than PD (Skrivankova et al., 2021).

This study investigates immune cell phenotypic characteristics that could assist researchers in identifying potential drug targets. For instance, elevated CD27 expression on memory B cell subsets is associated with an increased risk of Crohn’s disease; therapeutic targeting of CD27 on B cells may aid in reducing this risk (Zheng et al., 2017). Secondly, this study facilitates the development of personalized prevention and treatment strategies for PD: immune cell phenotypes, reflecting individual variations, enable the identification of individuals at elevated risk of the disease. Initiating interventions or targeted treatments at an early stage can significantly enhance an individual’s quality of life. For instance, individuals exhibiting low HLA-DR expression on monocytes, who are at a heightened risk for PD, could benefit from early lifestyle modifications, including changes in diet, increased exercise, and minimizing exposure to environmental hazards, to mitigate PD risk. This study’s primary advantage is its position as the most extensive genetic analysis conducted thus far in exploring the causal connection between immune cell phenotypes and PD. Unlike previous research focusing on a single cell type (Jensen et al., 2021) or limited immune cell phenotypes (Tian et al., 2022), our study encompasses a broad range of immune cell types.

This study has certain limitations. Firstly, our data selection was limited to studies measuring immune cell phenotypes in blood samples, despite evidence suggesting elevated levels in CSF samples of PD patients (Guan et al., 2022). However, this approach offers significant insight into the role of peripheral immunity in PD. Secondly, while two-sample Mendelian randomization offers a robust approach for causal inference, further validation via randomized controlled trials and animal studies is essential. Finally, the selection of immune cell data from Sardinians, due to the higher prevalence of certain immune-related genetic variants in Sardinia (Steri et al., 2017), necessitates validation of our study’s conclusions in broader population-based data.

## 5 Conclusion

Variants correlated with clinical outcomes frequently influence a multitude of immune phenotypes, rendering the identification of the specific immune phenotype genuinely associated with the disease process challenging. This study delves into the immune-related mechanisms of PD from the perspective of cellular subtypes, utilizing GWAS data analysis to identify immune phenotypes genuinely associated with PD. This facilitates the identification of potential druggable protein targets and lays a theoretical foundation for the development of future therapeutic strategies with immunomodulatory effects.

## Data availability statement

The raw data supporting the conclusions of this article will be made available by the authors, without undue reservation.

## Ethics statement

Ethical approval was not required for the study involving humans in accordance with the local legislation and institutional requirements. Written informed consent to participate in this study was not required from the participants or the participants’ legal guardians/next of kin in accordance with the national legislation and the institutional requirements.

## Author contributions

ZS: Writing – original draft. WL: Writing – original draft. YH: Writing – original draft. YX: Writing – original draft. HD: Formal analysis, Writing – original draft. YW: Writing – review & editing.
